# Artificial Selection Drives SNPs of Olfactory Receptor Genes into Different Working Traits in Labrador Retrievers

**DOI:** 10.1155/2022/8319396

**Published:** 2022-02-02

**Authors:** Min Yang, Han-Xin Zhang, Guang-Jun Geng, Fu-Jin Wang, Cheng-Wu Liu, Jian-Li Liu

**Affiliations:** ^1^Police-Dog Technology Department, Criminal Investigation Police University of China, Shenyang, Liaoning 110034, China; ^2^Mark Inspection Department, Criminal Investigation Police University of China, Shenyang, Liaoning 110854, China; ^3^Technology Department, Shenyang Traffic Police Detachment, Shenyang, Liaoning 110001, China; ^4^Laboratory Animal Center, Dalian Medical University, Dalian, Liaoning 116044, China; ^5^School of Life Science, Liaoning University, Shenyang, Liaoning 110036, China

## Abstract

Labs as guide dogs or sniffer dogs in usage have been introduced into China for more than 20 years. These two types of working dogs own blunt or acute olfactory senses, which have been obtained by artificial selection in relatively closed populations. In order to attain stable olfactory attributes and meet use-oriented demands, Chinese breeders keep doing the same artificial selection. Though olfactory behavior is canine genetic behavior, genotypes of OR genes formed by breeding schemes are largely unknown. Here, we characterized 26 SNPs, 2 deletions, and 2 insertions of 7 OR genes between sniffer dogs and guide dogs in order to find out the candidate alleles associated with working specific traits. The results showed that there were candidate functional SNP alleles in one locus that had statistically severely significant differences between the two subpopulations. Furthermore, the levels of polymorphism were not high in all loci and linkage disequilibrium only happened within one OR gene. Hardy–Weinberg equilibrium (HWE) tests showed that there was a higher ratio not in HWE and lower FST within the two working dog populations. We conclude that artificial selection in working capacities has acted on SNP alleles of OR genes in a dog breed and driven the evolution in compliance with people's intentions though the changes are limited in decades of strategic breeding.

## 1. Background

Artificial selection has been largely responsible for breed-specific and strain-specific traits of modern dogs [1]. Compared with early domestication of a dog breed driven by natural factors such as dietary conditions and geographical environments, the recent subpopulation differentiations are more dramatically caused by artificial goal-directed breeding practices, especially during the last two centuries [2]. Strain-dependent differences of dogs are mainly presented in body size, coat color, and work capacity [3, 4]. Labrador Retriever (Lab) is a traditional waterdog in Newfoundland. During the latter half of the nineteenth century, British breeders had refined and standardized Labs that had become a popular breed because they were famously friendly and exuberant around the world [5–7]. Many Labs play an important role such as sniffer dogs or guide dogs in many countries. These two types of work abilities are based on either enhanced olfaction or blunt olfaction. In order to attain stable olfactory attributes and meet use-oriented demands, Labs are bred in relatively closed populations by strict selection [8, 9].

The offsprings of pure breed Labs as guide dogs or sniffer dogs abroad were purchased from Japan by guide dogs training organizations and from Germany by police departments into China two decades ago. The same as foreign ones did, Chinese working dog organizations continued to breed Labs following the principle of closed-flock reproduction and occasionally adopted some excellent bloodlines of Labs from amateurs in the civilian clubs into Lab working dogs populations. Based on Labs' health, a temperament test of scent interest and a search test divide Labs into candidates of guide dogs with no scent interest or sniffer dogs displaying great scent interest and eager searching during early training [10–12]. Then, behavioral suitability of a working dog is shaped by specialized training [8, 13]. Subsequently, the olfactory detection phenotype of qualified sniffer dogs is that the dog indicates a target odor in the manner in which it was trained while qualified guide dogs could navigate by avoiding obstacles [14–16]. As far as disqualified Labs are concerned, their outcome is to be screened out of the subpopulations. Therefore, the offsprings did not degenerate and gradually became more competent for mobility support for the visually impaired/blind or scent detection for law implement. Detailed subgroup analyses are expected to explain the genetic basis for variation in olfactory behavior and to establish protocols for the reproduction of the two Labs' working strains.

Olfactory capacity and sensitivity are related to canine olfactory receptor gene polymorphism in dog breeds and individuals [9, 13]. Selections of different forms have acted on OR genes in dogs since domestication. The genetic diversity of 109 OR genes in six breeds including Labs was studied, which were representative of a large number of families, subfamilies, and clusters [17]. Through OR genes sequencing data analysis, the SNPs were identified and its Minor Allele Frequency (MAF) values testified that the 8 SNPs in 7 OR genes were private and restricted to only Labs. Because most of private SNPs arose following dog-breed formation under the selection from breeding practices and SNP alleles might present a pair of phenotypes' pros and cons in olfactory cognition, 30 SNPs including 7 private SNPs and 23 representative SNPs in the same OR genes might be studied to find out how the artificial selection drives them into phenotypic differentiation of olfactory behavior in Labs. Identifying the distributions of SNP alleles between the two subgroups might provide insights into genetic variation that might explain specific working traits and then efficiently obtain a desired phenotype [18].

## 2. Materials and Methods

### 2.1. Ethical Statement

This study was carried out in Police-Dog Technology Department, Criminal Investigation Police University of China. The protocol for the animal experiment was reviewed and approved by the Ethics Committee of Police-Dog Technology Department (JQJS2019-0001), and all applicable institutional and governmental regulations concerning the ethical use of animals were followed.

### 2.2. Animals and Blood Sample Collection

The trial included two subpopulations, 49 guide dogs (males, *n* = 36; females, *n* = 13) and 43 sniffer dogs (males, *n* = 31; females, *n* = 12) of Labs, whose ages ranged from 2 to 4 years old. The Labs were reproduced by China Guide Dog Training Center and Police-Dog Technology Department, Criminal Investigation Police University of China. According to the certificates of the dogs' pedigree, the two groups sampled were unrelated. All the dogs treated in a humane manner passed the qualification assessments of working dogs and had been competent for the tasks assigned. Blood samples were obtained from each dog, and then Universal Genomic DNA Extraction Kit (Takara, China) was used to extract DNAs.

### 2.3. SNP and Primer Design

30 SNP loci lying in exonic regions of 7 OR genes were analyzed and are shown in Table 1. Asterisks marked out the breed-specific SNPs [17]. No asterisks meant that the SNPs could be found in other dog breeds besides Labs. Because non-breed-specific SNPs in the 7 OR genes led to amino-acid substitutions, frameshift variants, protein altering variants, and splicing donor variants, they were studied too. Pairs of specific primers for fraction amplification of 7 OR genes were designed using the Primer-BLAST and Primer Premier 5 program. The primer sequences, fragment sizes, and annealing temperatures (Tm) above mentioned are shown in Table S1.

### 2.4. PCR and Sequencing

Polymerase chain reaction (PCR) was conducted using a Veriti thermal cycler (ABI, USA) in a total volume of 50 *μ*L that included 2 *μ*L 100 ng of genomic DNA, 25 *μ*L Premix Taq (Takara, China), and 1 *μ*M (final conc.) of each specific primer and water up to 50 *μ*L. PCR products were loaded onto a 2% agarose gel stained with GeneFinder (Biov, China) and DNAs were extracted from the gel. The purified products of PCR were cloned into pMD18-T vector (Takara, China) and positive clones specific for each of the seven amplicons were sequenced in both directions. Nucleotide BLAST of the sequences was done. Then the purified products of PCR were massively directly sequenced by the forward or reverse primers.

Sequencing PCR reaction was performed in a 10 *μ*l reaction system that consisted of Premix 4 *μ*L, the purified PCR products 0.5 *μ*L, primer 1 *μ*L (1 *μ*M), and 4.5 *μ*L ddH_2_O, starting with 30 cycles of denaturation at 96°C for 20 secs, annealing at 55°C for 20 secs, and extension at 60°C for 4 mins, followed by an extra stop reaction initiated by adding 0.5 *μ*L of glycogen, 1 *μ*L of 3M sodium acetate (pH5.2), and 1 *μ*L of 100 mM Na_2_-EDTA (pH8.0) per reaction. 40 *μ*L of sample loading solution was added into DNA precipitates with ethanol in individual tubes to resuspend the samples. The capillary electrophoresis was analyzed on Beckman GeXP Genetic Analysis System according to Genome-Lab Dye Terminator Cycle Sequencing with Quick Start Kit (Beckman, USA).

### 2.5. Statistical Analysis

Allele frequency, Genotype frequency, observed heterozygosity (Ho), and polymorphism information content (PIC) were calculated by PowerStats software v1.2 [19]. Expected heterozygosity (He) was counted by CERVUS 3.0.3 [20] software. The *F*-value of an inbreeding coefficient was derived by [1- (Ho/He)] by EXCEL software too [21]. Hardy–Weinberg equilibrium (HWE) tests were performed by ARLEQUIN software v3.5 [22]. Linkage disequilibrium analyses (correlation coefficient, *r*^2^) and the chi-square test about alleles on single locus in a pairwise study were analyzed by SHEsis online tool. A logistic regression analysis of alleles and genotypes was performed on IBM SPSS Statistics 25 software. Fst values were calculated by GenAlEx 6.5 software [23]. The tools including Protein Variation Effect Analyzer (PROVEAN) http://provean.jcvi.org/index.php [24], Align-Grantham variation and Grantham deviation (Align GVGD) http://agvgd.hci.utah.edu/ [25], and Sorting Intolerant from Tolerant (SIFT) https://sift.bii.astar.edu.sg/ [26] were used to predict the effect of nonsynonymous SNPs in the encoded proteins.

## 3. Results

Allele frequencies and genotype frequencies of 30 SNP loci are given in Table 2, which ranged from 0 to 1. Overall, the most of the SNP loci had a major allele whose frequencies were beyond 0.8 except for six loci in guide dogs, seven loci in sniffer dogs, and seven loci in the collective. Furthermore, a unique homozygous genotype was presented in 12 SNP loci, while the only heterozygous genotype was found in 2 SNP loci within all Labs. So, the 14 SNP loci were excluded in the chi-square test for differences of alleles on a single SNP locus in a pairwise study. Among the 12 homozygous loci, the deletions of the loci OR16C11:c.633del and OR7215:c.398del that could result in no expression of gene OR16C11 and a splice donor variant of gene OR7215 did not happen and the insertion of OR7215:c.272ins producing a protein altering variant did not happen either. Noticeably the insertion of OR7215:c.273insCTTCCA producing a frameshift variant occurred to all Labs. Regarding the remaining 16 loci, there were differences either in genotypes or in genotype frequencies between sniffer dogs and guide dogs. All three genotypes were found in the two loci OR0007:c.691 G > C and OR0007:c.830 C >T only in guide dogs while sniffer dogs presented GG or GC genotype in the locus OR0007:c.691 G > C and were all homozygotes with TT genotype in the locus OR0007:c.830 C >T. Consequently, the alleles in seven SNP loci showed significant differences (*P* < 0.05), and highly significant differences (*P* < 0.001) were shown in the four SNP loci of which the three SNP loci were breed-specific between the two working groups given in Table 3. On the basis of differences of the SNP alleles between sniffer dogs and guide dogs, a binary logistic regression analysis was carried out, and it found that the alleles and genotypes in five SNP loci could affect types of working dogs (*P* < 0.05) given in Table 4.

In this study, except for 12 homozygous monogenotype loci, PIC values of 18 SNPs ranged from 0 to 0.375. Among the 18 SNPs, 10 SNPs belonged to low polymorphism (PIC<0.25) and 5 SNPs belonged to moderate polymorphism (0.25 < PIC<0.5) for all Labs. As far as the other three SNPs, OR0007:c.331 T >C, OR0007:c.430 G > A, and OR7215:c.251 C >T, were concerned, they belonged to either low or moderate polymorphism, which differed between guide dogs and sniffer dogs (seen in Table 5). As could be seen in Table 5, Ho values were within the wide scope of 0 to 1 and He values were not more than 0.5. There were unanimously low Ho values in 12 SNP loci (<0.5) and high Ho values in 4 SNP loci (≥0.5) in the two working groups except that the values varied in the SNP loci of OR0007:c.331 T >C and OR0007:c.829C>T between guide dogs and sniffer dogs.

*P* values of HWE tests for 18 SNP loci were given in Table 5. The total number of genotyped Labs was 92 and so HWE *P* = 0.0005 emphasized the need for multiple comparisons by applying Bonferroni correction (the significant level = 0.05/92). Except 12 homozygous monogenotype loci, the results revealed that 5 SNP loci were out of HWE (*P* < 0.0005) and 5 SNP loci were in the state of Hardy–Weinberg balance (*P* > 0.0005) for all Labs. The other eight SNP loci showed either significant departure or no significant deviations from HWE between working populations, while six of the eight SNP loci were in HWE in the collective. After the two working groups and the collective were analyzed, respectively, there was a higher ratio 73.3% not in HWE in sniffer dogs than 66.7% in guide dogs and 63.3% in the collective. Furthermore, the value of genetic differentiation between the two working populations was 0.054 (0.05﹤FST﹤0.15) when the loci OR0006:c.524 G > T, OR0007:c.331 T >C, OR0007:c.430 G > A, OR0007:c.793 G > C, and OR16C11:c.294 G > A in HWE were considered.

**Figure 1:**
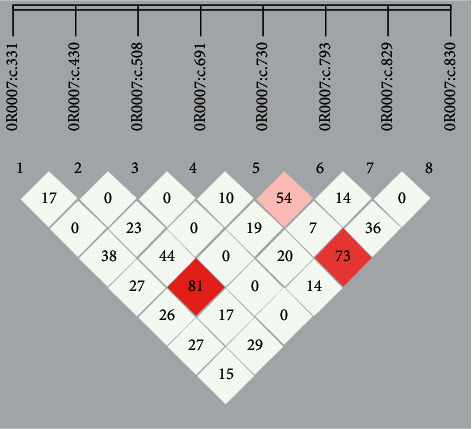
Linkage disequilibrium analyses were performed between pairwise SNP loci in OR0007 gene in the two working populations of Labs. Actual correlation coefficient (*r*^2^) values were magnified 100 times in the small box.

The *F*-values of average inbreeding coefficient were −0.226 for guide dogs and −0.241 for sniffer dogs given in Table 6. Linkage disequilibrium values for OR0007 gene, OR16C11 gene, DOPRX09 gene, and OR7215 gene were calculated for every working dog population. The results displayed that the *r*^2^ value that was more than 0.80 was shown only within OR0007 gene for the two working dog groups (see Figure 1).

Nonsynonymous mutations are divided into two kinds of being tolerated (>0.05) and deleterious (≤ 0.05) with SIFT. The biological impact effects of amino acid substitution induced by nonsynonymous SNPs were considered “deleterious” (<−2.5) and “neutral” (>−2.5) by PROVEAN v.1.1.3. We estimated 24 nonsynonymous SNPs using SIFT, PROVEAN, and Align GVGD. 6 SNPs were predicted to be “untolerated” with a score below 0.05 by SIFT, 7 SNPs are predicted to be “deleterious” by PROVEAN and 10 SNPs were classified as a class C65 change, the first highest pathogenicity category by Align GVGD (see Table 7). OR0006:c.524 G > T, OR16C11:c.368 C >A, OR7215:c.251 C >T, and OR04B06 : 215T >G were considered to affect protein function or structure with all of the three analysis software.

## 4. Discussion

The aim of this study was to explain how use-oriented selection could lead to phenotypic changes of olfactory behavior in Labs. Olfactory traits of guide Labs and sniffer Labs are expected to play a role in canine behavioral genetics [27]. We focused on 26 SNPs, 2 deletions, and 2 insertions in 7 OR genes from the two Labs working dog populations. The genotyping test found that one population presented only one type of homozygote compared with the other population presenting two or three genotypes in 7 loci, suggesting that the alleles were purified and needed for working traits of guide dogs and sniffer dogs by rapid evolution [3]. These regions of extended homozygosity might be best explained by selection rather than genetic drift [28]. On the contrary, heterozygotes or one type of homozygote in a certain locus could occur in any working dog group, which indicated that putative SNP alleles and genotypes were chosen and possibly contributed to the common working traits under artificial selection. The significant differences between sniffer dogs and guide dogs happened to 36.7% of these loci, which explained that behavioural phenotypes in a dog breed might be affected by genetic background and controlled by multiple SNP alleles with large effects, which were preserved by artificial selection. So, special alleles might play functional roles that shaped working dog traits within a dog breed [28]. Moreover, functional loss or alternation of OR genes resulted from deleterious mutations could have been avoided through maintaining a large population size that enabled the effects of genetic drift to be negligible [29, 30]. Because the differences of these alleles were analyzed in small volume samples and the association between the alleles and olfactory behavior phenotypes was not analyzed, their effects remained potential.

Meanwhile, the loss of genomic diversity (PIC<0.375) resulted from strong selective pressures of olfactory behavior through gene pathways [31]. In addition, the data sets that Ho values were higher than He values except the locus OR7215:c.578 A *>T* in any Labs group showed that selective breeding in working dog populations had tried to gain popular hereditary features and avoid inbreeding in a limited degree. Furthermore, the mean *F*-values of inbreeding coefficients verified the negative regression in the two working dog groups. Low levels of linkage disequilibrium obtained for every working dog subpopulation were consistent with homogeneity within a dog breed [32, 33]. This highlighted that the most of SNP alleles within individual OR genes were not inherited as a block and suggested an ongoing gene conversion process for desirable working traits although it took people about 150 years to develop a dog breed.

The analysis of 26 SNPs by the collective and two working Labs groups showed that the proportions of being in HWE were lower than 52.6% in a former study of Labs that was a lower proportion among dog breeds [34]. It was a possible explanation that the selection of desirable phenotypic traits depending on alleles had screened the disqualified Labs out of the populations. Consequently, the artificial selection concurrently indirectly caused assortative mating and worsened the state of being out of HWE since a large number of working dogs had not made up for the shortcoming of olfactory behavior selection thoroughly in the relatively closed populations. The degree of genetic differentiation (Fst) between the two working Labs groups was 0.054, a little higher than the borderline 0.05 above which a dog breed showed moderate differentiation among its subpopulations [35]. An important reason was that breeders introduced ideal sires and dams from other Labs' subpopulations which had the improvement of some working characteristics instead of some other dog breeds with very close genetic relationship.

In silico prediction tools, SIFT, PROVEAN, and Align GVGD evaluated the pathogenicity of missense variants of 24 SNPs because bioinformatics analysis alone was usually not sufficient [36]. In order to decrease the proportion of false positive results, the positive judgments in agreement with each other were accepted. So the four missense variants residing in EC1, EC2, and TM2 domains of OR protein were not tolerated on the basis of the comprehensive analysis. Substituting amino acids by missense mutations in OR genes could contribute to increase or decrease in the sensitivity towards target odorants [37]. Among them, OR16C11:p.A123D residing in EC1 domain showed a significant difference and OR7215:p.P84 L residing in TM2 domain showed a severely significant difference between the two working Labs groups. In addition, OR7215:p.P84 L showed a possible functional role in typing working Labs subpopulations. So the functional allele is possibly related to working capacities of Labs, which might help breeders to pick out Labs of different levels in olfactory acuity early and accurately and set up different working Labs' strains. But the similar study is not still carried out in other sniffer dogs, for example, Springer Spaniels and bloodhounds. Additionally, the functions of these alleles might be examined by the transduction of the olfactory message in the future [38].

## 5. Conclusion

A number of analyses were conducted to reveal canine breed phylogeny or relation with some complicated qualities due to SNP variation. However, few studies aimed at subpopulation differentiations within a same dog breed. The strong artificial selection was only performed urgently to gain working attributes. Labs working in different fields acted as the sample model. SNP loci in OR genes presented indexes on the positive and negative selection, namely, a preference for acute sniffing and being numb about odor. Our analysis demonstrated that possibly functional alleles were quickly evolving in compliance with people's intentions though the changes were limited in decades of artificial selection.

## Figures and Tables

**Table 1:** Characteristics of the analyzed SNPs.

SNP	Amino acid variant	Domain	MAF
OR0006:c.524 G > *T*^*∗*^	p.R175L	EC2	<0.01
OR0007:c.331T>C	p.F111L	TM3	0.50
OR0007:c.430 G > *A*	p.G144S	IC2	0.50
OR0007:c.508 G > *A*^*∗*^	p.V170M	TM4	0.01
OR0007:c.691 G > *C*	p.V231L	TM5	0.38
OR0007:c.730 T *>C*	p.W244R	TM6	0.39
OR0007:c.793 G > *C*	p.V265L	TM6	0.41
OR0007:c.829 C *>T*	p.P277S	EC3	0.40
OR0007:c.830 C *>T*	p.P277L	EC3	0.40
OR16C11:c.294 G > *A*^*∗*^	p.Q98Q	TM2	0.03
OR16C11:c.368 C *>A*	p.A123D	EC1	0.12
OR16C11:c.391 A *>G*	p.T131A	EC1	0.01
OR16C11:c.632del	I212Y Ter2	TM4	—
DOPRX09:c.479 G > *A*	p.S160N	EC2	0.49
DOPRX09:c.481 T *>G*	p.S161A	EC2	0.49
DOPRX09:c.486 T *>G*	p.F162L	TM5	0.49
DOPRX09:c.889 G > *A*^*∗*^	p.V263I	C	0.03
OR08G02:c.756 G > *T*^*∗*^	p.G252G	N/A	—
OR7215:c.251 C *>T*	P.84L	TM2	0.36
OR7215:c.272ins- > TCTTCC	p.C91FFR	EC1	—
OR7215:c.273ins-> CTTC/CTTCCA	p.C91CFX91CFH	EC1	0.25
OR7215:c.286 G > *A*	p.G96S	EC1	0.02
OR7215:c.299 A *>G*	p.Y100C	TM3	0.21
OR7215:c.339 G > *A*	p.M113I	TM3	0.14
OR7215:c.398delTGCGCTA > *-*	—	TM4	0.11
OR7215:c.413 G > *A*^*∗*^	R138H	TM4	0.04
OR7215:c.578 A *>T*^*∗*^	N193I	TM5	—
OR04B06:c.215 T *>G*	L72 R	TM2	—
OR04B06:c.394 A *>G*	A132 T	TM4	—
OR04B06:c.411 A *>T*	F137 L	TM4	—

^*∗*^Asterisks marked out the breed-specific SNPs. No asterisks meant that the SNPs could be found in other dog breeds besides Labs. MAF: minor allele frequency.

**Table 2:** Allele frequency and genotype frequency of 30 SNP loci.

Loci	Guide dog						Sniffer				Collective						
	Allele frequency		Genotype frequency				Allele frequency		Genotype frequency		Allele frequency		Genotype frequency				
OR0006:c.524 G > *T* ^*∗*^	G/0.980	T/0.020	GG/0.959		GT/0.041		G/0.942	T/0.058	GG/0.884	GT/0.116	G/0.962	T/0.038	GG/0.924		GT/0.076		
OR0007:c.331 T *>C*	T/0.878	C/0.122	TT/0.755		TC/0.245		T/0.709	C/0.291	TT/0.419	TC/0.581	T/0.799	C/0.201	TT/0.598		TC/0.402		
OR0007:c.430 G > *A*	A/0.796	G/0.204	AA/0.592		GA/0.408		A/0.942	G/0.058	AA/0.884	GA/0.116	A/0.864	G/0.136	AA/0.728		GA/0.272		
OR0007:c.508 G > *A* ^*∗*^	G/1		GG/1				G/0.907	A/0.093	GG/0.814	GA/0.186	G/0.957	A/0.043	GG/0.913		GA/0.087		
OR0007:c.691 G > *C*	G/0.663	C/0.337	GG/0.408	GC/0.510		CC/0.082	G/0.628	C/0.372	GG/0.256	GC/0.744	G/0.647	C/0.353	GG/0.337	GC/0.620			CC/0.043
OR0007:c.730 T *>C*	C/0.878	T/0.122	CC/0.755		CT/0.245		C/1		CC/1		C/0.935	T/0.065	CC/0.870		CT/0.130		
OR0007:c.793 G > *C*	C/0.837	G/0.163	CC/0.673		CG/0.327		C/0.942	G/0.058	CC/0.884	CG/0.116	C/0.886	G/0.114	CC/0.772		CG/0.228		
OR0007:c.829 C *>T*	C/0.541	T/0.459	CC/0.082		TC/0.918		C/0.5	T/0.5	TC/1		C/0.522	T/0.478	CC/0.043		TC/0.957		
OR0007:c.830 C *>T*	C/0.837	T/0.163	TT/0.755	TC/0.163		CC/0.082	T/1		TT/1		C/0.087	T/0.913	TT/0.870	TC/0.087		CC/0.043	
OR16C11:c.294 G > *A* ^*∗*^	G/0.929	A/0.071	GG/0.857		GA/0.143		G/0.884	A/0.116	GG/0.767	GA/0.233	G/0.908	A/0.092	GG/0.815		GA/0.185		
OR16C11:c.368 C *>A*	C/1		CC/1				C/0.953	A/0.047	CC/0.907	CA/0.093	C/0.978	A/0.022	CC/0.957		CA/0.043		
OR16C11:c.391 A *>G*	A/0.969	G/0.031	AA/0.939		GA/0.061		A/1		AA/1		A/0.984	G/0.016	AA/0.967		GA/0.033		
OR16C11:c.632del	NONE						NONE				NONE		NONE				
DOPRX09:c.479 G > *A*	G/0.5	A/0.5	GA/1				G/0.5	A/0.5	GA/1		G/0.5	A/0.5	GA/1				
DOPRX09:c.481 T *>G*	T/0.5	G/0.5	TG/1				T/0.5	G/0.5	TG/1		T/0.5	G/0.5	TG/1				
DOPRX09:c.486 T > *G*	G/0.541	T/0.459	GG/0.082		GT/0.918		G/0.698	T/0.302	GG/0.395	GT/0.605	G/0.614	T/0.386	GG/0.228		GT/0.772		
DOPRX09:c.889 G > *A*∗	G/0.102	A/0.898	GG/0.796		GA/0.204		G/1		GG/1		G/0.946	A/0.054	GG/0.891		GA/0.109		
OR08G02:c.756 G > *T* ^*∗*^	G/1		GG/1				G/1		GG/1		G/1		GG/1				
OR7215:c.251 C *>T*	C/0.163	T/0.837	CC/0.163		TT/0.837		C/0.605	T/0.395	CC/0.605	TT/0.395	T/0.370	T/0.630	CC/0.370		TT/0.630		
OR7215:c.272ins	None						None				None						
OR7215:c.273ins	CTTCCA/1		CTTCCA/1				CTTCCA/1		CTTCCA/1		CTTCCA/1		CTTCCA/1				
OR7215:c.286 G > *A*	G/1		GG/1				G/1		GG/1		G/1		GG/1				
OR7215:c.299 A *>G*	G/1		GG/1				G/1		GG/1		G/1		GG/1				
OR7215:c.339 G > *A*	G/1		GG/1				G/1		GG/1		G/1		GG/1				
OR7215:c.398del	None						None				None						
OR7215:c.413 G > *A* ^*∗*^	G/1		GG/1				G/1		GG/1		G/1		GG/1				
OR7215:c.578 A *>T* ^*∗*^	A/1		AA/1				A/0.838	T/0.162	AA/0.838	TT/0.162	A/0.924	T/0.076	AA/0.924		T T/0.076		
OR04B06:c.215 T *>G*	T/1		TT/1				T/1		TT/1		T/1		TT/1				
OR04B06:c.394 A *>G*	G/1		GG/1				G/1		GG/1		G/1		GG/1				
OR04B06:c.411 A *>T*	T/1		TT/1				T/1		TT/1		T/1		TT/1				

^*∗*^Asterisks marked out the breed-specific SNPs. No asterisks meant that the SNPs could be found in other dog breeds besides Labs.

**Table 3:** Differences of SNP alleles between sniffer dogs and guide dogs.

Loci	*P* value
OR0006:c.524 G > *T*^*∗*^	0.182
OR0007:c.331 T *>C*	0.005
OR0007:c.430 G > *A*	0.004
OR0007:c.508 G > *A*^*∗*^	0.002
OR0007:c.691 G > *C*	0.617
OR0007:c.730 T > *C*	0.001
OR0007:c.793 G > *C*	0.025
OR0007:c.829 C > *T*	0.580
OR0007:c.830 C > *T*	8.88*e* − 005
OR16C11:c.294 G > *A*^*∗*^	0.295
OR16C11:c.368 C > *A*	0.031
OR16C11:c.391 A > *G*	0.183
DOPRX09:c.486 T > *G*	0.029
DOPRX09:c.889 G > *A*^*∗*^	0.002
OR7215:c.251 C > *T*	6.25*e* − 010
OR7215:c.578 A > *T*^*∗*^	3.28*e* − 005

Asterisks marked out the breed-specific SNPs. No asterisks meant that the SNPs could be found in other dog breeds besides Labs.

**Table 4:** Logistic regression analysis of alleles and genotypes between sniffer dogs and guide dogs.

Loci	Allele		Genotype	
	OR (95% CI)	*P*	OR (95% CI)	*P*
OR0007:c.331 T *>C*	3.106(1.453–6.636)	0.003	4.282(1.760–10.422)	0.001
OR0007:c.430 G > *A*	5.256(1.720–16.068)	0.004	5.241(1.758–15.631)	0.003
OR0007:c.508 G > *A*^*∗*^	2.030 × 10^9^(0.000-)	0.999	2.262 × 109(0.000-)	0.999
OR0007:c.730 T *>C*	1.615 × 10^9^(0.000-)	0.999	1.877 × 109(0.000-)	0.999
OR0007:c.793 G > *C*	8.195(1.827–36.770)	0.006	3.685(1.218–11.150)	0.021
OR0007:c.830 C *>T*	0.000(0.000-)	0.996	(1) 0.000(0.000-)	(1) 0.999
			(2) 0.000(0.000-)	(2) 0.999
OR16C11:c.368 C *>A*	1.931 × 10^9^(0.000-)	0.999	2.030 × 109(0.000-)	0.999
DOPRX09:c.486 T *>G*	1.880(1.023–3.455)	0.042	7.36(2.235–24.213)	0.001
DOPRX09:c.889 G > A^*∗*^	0.000(0.000-)	0.996	0.000(0.000-)	0.999
OR7215:c.251 C *>T*	7.838(3.938–15.601)	0.000	7.838(2.961–20.748)	0.000
OR7215:c.578 A *>T*∗	0.000(0.000-)	0.998	0.000(0.000-)	0.999

Asterisks marked out the breed-specific SNPs. No asterisks meant that the SNPs could be found in other dog breeds besides Labs.

**Table 5:** Genetic polymorphism and HWE analysis of the collective and working dog subpopulations.

Loci	Ho			He			PIC			HWE		
	Guide	Sniffer	Collective	Guide	Sniffer	Collective	Guide	Sniffer	Collective	Guide	Sniffer	Collective
OR0006:c.524 G > *T*^*∗*^	0.041	0.116	0.076	0.039	0.109	0.073	0.038	0.103	0.070	0.884	0.686	0.704
OR0007:c.331 T > *C*	0.245	0.581	0.402	0.214	0.413	0.321	0.191	0.328	0.270	0.329	0.007	0.016
OR0007:c.430 G > *A*	0.408	0.116	0.272	0.325	0.109	0.235	0.272	0.103	0.207	0.073	0.686	0.132
OR0007:c.508 G > *A*∗	0	0.186	0.087	0	0.169	0.082	0	0.154	0.079	—	0.501	0.663
OR0007:c.691 G > *C*	0.510	0.744	0.620	0.447	0.467	0.457	0.347	0.358	0.352	0.320	1*E* − 04	6*E* − 04
OR0007:c.730 T > *C*	0.245	0	0.130	0.225	0	0.122	0.191	0	0.114	0.329	—	0.503
OR0007:c.793 G > *C*	0.327	0.116	0.228	0.273	0.109	0.202	0.236	0.103	0.182	0.172	0.686	0.217
OR0007:c.829 C > *T*	0.918	0	0.957	0.497	0	0.499	0.373	0.375	0.375	3*E* − 09	5*E* − 11	1*E* − 18
OR0007:c.830 C > *T*	0.163	0	0.087	0.273	0	0.159	0.236	0	0.146	0.005	—	1*E* − 05
OR16C11:c.294 G > *A*∗	0.143	0.233	0.185	0.132	0.205	0.167	0.123	0.184	0.153	0.590	0.388	0.329
OR16C11:c.368 C > *A*	0	0.093	0.043	0	0.090	0.043	0	0.086	0.042	—	0.749	0.831
OR16C11:c.391 A > *G*	0.061	0	0.033	0.060	0	0.031	0.058	0	0.031	0.825	—	0.874
DOPRX09:c.479 G > *A*	1	1	1	0.5	0.5	0.5	0.375	0.375	0.375	3*E* − 12	5*E* − 11	9*E* − 22
DOPRX09:c.481 T > *G*	1	1	1	0.5	0.5	0.5	0.375	0.375	0.375	3*E* − 12	5*E* − 11	9*E* − 22
DOPRX09:c.486 T > *G*	0.918	0.605	0.772	0.497	0.422	0.474	0.373	0.333	0.362	3*E* − 09	0.004	2*E* − 09
DOPRX09:c.889 G > *A*∗	0.204	0	0.109	0.183	0	0.102	0.166	0	0.097	0.426	—	0.581
OR7215:c.251-C > *T*	0	0	0	0.273	0.472	0.466	0.236	0.360	0.358	3*E* − 12	5*E* − 11	9*E* − 22
OR7215:c.578-A > *T*^*∗*^	0	0	0	0	0.273	0.140	0	0.236	0.131	—	5*E* − 11	9*E* − 22

Asterisks marked out the breed-specific SNPs. No asterisks meant that the SNPs could be found in other dog breeds besides Labs. Observed heterozygosity (Ho), expected heterozygosity (He), polymorphism information content (PIC), and Hardy–Weinberg equilibrium (HWE) values.

**Table 6:** *F*-value of an inbreeding coefficient in sniffer dogs and guide dogs.

Population	Num	*F*-value	SE
Guide	49	−0.226	0.038
Sniffer	43	−0.241	0.044

**Table 7:** Prediction of the deleterious effect of the nonsynonymous polymorphism in OR genes by SIFT, PROVEAN, and Align GVGD.

SNP	Amino acid variant	SIFT	PROVEAN	Align GVGD		
				GV	GD	Prediction
OR0006:c.524 G > *T*∗	p.R175 L	0	−6.292	0.00	101.88	Class C65
OR0007:c.331 T > *C*	p.F111 L	0.41	−2.834	0.00	21.82	Class C15
OR0007:c.430 G > *A*	p.G144S	1	2.354	0.00	55.27	Class C55
OR0007:c.508 G > *A*∗	p.V170 M	0.07	−1.330	0.00	21.52	Class C15
OR0007:c.691 G > *C*	p.V231 L	1	1.909	0.00	31.78	Class C25
OR0007:c.730 T *>C*	p.W244 R	1	3.875	0.00	101.29	Class C65
OR0007:c.793 G > *C*	p.V265 L	1	2.075	0.00	31.78	Class C25
OR0007:c.829 C *>T*	p.P277S	1	0.799	0.00	73.35	Class C65
OR0007:c.830 C > *T*	p.P277 L	0.13	4.918	0.00	97.78	Class C65
OR16C11:c.368 C *>A*	p.A123D	0	−5.989	0.00	125.75	Class C65
OR16C11:c.391 A *>G*	p.T131 A	0.19	−2.605	0.00	58.02	Class C55
DOPRX09:c.479 G > *A*	p.S160 N	0.11	−0.423	0.00	46.24	Class C45
DOPRX09:c.481 T *>G*	p.S161 A	0.06	−0.009	0.00	99.13	Class C65
DOPRX09:c.486 T *>G*	p.F162 L	1	−1.589	0.00	21.82	Class C15
DOPRX09:c.889 G > *A*∗	p.V297I	0.02	−0.826	0.00	29.61	Class C25
OR7215:c.251 C *>T*	p.P84 L	0.01	−7.578	0.00	97.78	Class C65
OR7215:c.286 G > *A*	p.G96S	1	0.866	0.00	55.27	Class C55
OR7215:c.299 A *>G*	p.Y100 C	1	10.826	0.00	193.72	Class C65
OR7215:c.339 G > A	p.M113I	0.19	−1.904	0.00	10.12	Class C0
OR7215:c.413 G > *A*∗	p.R138H	0.01	−4.234	0.00	28.82	Class C25
OR7215:c.578A>T∗	p.N193I	N/A	−0.888	0.00	148.91	Class C65
OR04B06:215T>G	p.L72R	0	−5.605	0.00	101.88	Class C65
OR04B06:394A>G	p.A132T	0.08	−2.476	0.00	58.02	Class C55
OR04B06:411A>T	p.F137L	0.77	1.195	0.00	21.82	Class C15

Asterisks marked out the breed-specific SNPs. No asterisks meant that the SNPs could be found in other dog breeds besides Labs. SIFT scores range from 0 to 1. The amino acid substitution is predicted to be damaging if the score is ≤0.05 and tolerated if the score is > 0.05. N/A: not available, GD ≥ C65 = most likely affected, and GD ≥ C0 = less likely affected. The amino acid substitution is predicted to be deleterious (<−2.5) and neutral (>−2.5) by PROVEAN.

## Data Availability

The data used to support the findings of this study are included within the article and the supplementary information file.

## Supplementary Materials

Supplementary Materials
*Table S1*. Primer sequences of 7 OR genes.

## References

[1] Ostrander E. A., Wayne R. K., Freedman A. H., Davis B. W. (2017). Demographic history, selection and functional diversity of the canine genome. *Nature Reviews Genetics*.

[2] Lampi S., Donner J., Anderson H., Pohjoismäki J. (2020). Variation in breeding practices and geographic isolation drive subpopulation differentiation, contributing to the loss of genetic diversity within dog breed lineages. *Canine Medicine and Genetics*.

[3] Cagan A., Blass T. (2016). Identification of genomic variants putatively targeted by selection during dog domestication. *BMC Evolutionary Biology*.

[4] Plassais J., Kim J., Davis B. W. (2019). Whole genome sequencing of canids reveals genomic regions under selection and variants influencing morphology. *Nature Communications*.

[5] Asher L., Harvey N. D., Green M., England G. C. W. (2017). Application of survival analysis and multistate modeling to understand animal behavior: examples from guide dogs. *Frontiers in Veterinary Science*.

[6] Konno A., Inoue-Murayama M., Yabuta S. (2018). Effect of canine oxytocin receptor gene polymorphism on the successful training of drug detection dogs. *Journal of Heredity*.

[7] Shouldice V. L., Edwards A. M., Serpell J. A., Niel L., Robinson J. A. B. (2019). Expression of behavioural traits in goldendoodles and labradoodles. *Animals-Basel*.

[8] Harvey N. D., Craigon P. J., Blythe S. A., England G. C. W., Asher L. (2017). An evidence-based decision assistance model for predicting training outcome in juvenile guide dogs. *PLoS One*.

[9] Lesniak A., Walczak M., Jezierski T., Sacharczuk M., Gawkowski M., Jaszczak K. (2008). Canine olfactory receptor gene polymorphism and its relation to odor detection performance by sniffer dogs. *Journal of Heredity*.

[10] Arata S., Momozawa Y., Takeuchi Y., Mori Y. (2010). Important behavioral traits for predicting guide dog qualification. *Journal of Veterinary Medical Science*.

[11] Hunt R. L., England G. C. W., Asher L., Whiteside H., Harvey N. D. (2020). Concurrent and predictive criterion validity of a puppy behaviour questionnaire for predicting training outcome in juvenile guide dogs. *Animals*.

[12] Lazarowski L., Rogers B., Krichbaum S., Haney P., Smith J. G., Waggoner P. (2021). Validation of a behavior test for predicting puppies’ suitability as detection dogs. *Animals*.

[13] Yang M., Geng G. J., Zhang W., Cui L., Zhang H. X., Zheng J. L. (2016). SNP genotypes of olfactory receptor genes associated with olfactory ability in German Shepherd dogs. *Animal Genetics*.

[14] Bogaerts E., Moons C. P. H., Van Nieuwerburgh F., Peelman L., Saunders J. H., Broeckx B. J. G. (2019). Rejections in an non-purpose bred assistance dog population: reasons, consequences and methods for screening. *PLoS One*.

[15] Concha A., Mills D. S., Feugier A. (2014). Using sniffing behavior to differentiate true negative from false negative responses in trained scent-detection dogs. *Chemical Senses*.

[16] Gaunet F., Besse S. (2019). Guide dogs’ navigation after a single journey: a descriptive study of path reproduction, homing, shortcut and detour. *PLoS One*.

[17] Robin S., Tacher S., Rimbault M. (2009). Genetic diversity of canine olfactory receptors. *BMC Genomics*.

[18] Friedenberg S. G., Meurs K. M., Mackay T. F. (2016). Evaluation of artificial selection in Standard Poodles using whole-genome sequencing. *Mammalian Genome : Official Journal of the International Mammalian Genome Society*.

[19] Wang M. L., Jin X. Y., Xiong X. (2019). Polymorphism analyses of 19 STRs in Labrador Retriever population from China and its heterozygosity comparisons with other retriever breeds. *Molecular Biology Reports*.

[20] Kalinowski S. T., Taper M. L., Marshall T. C. (2007). Revising how the computer program CERVUS accommodates genotyping error increases success in paternity assignment. *Molecular Ecology*.

[21] Pedersen N. C., Pooch A. S., Liu H. W. (2016). A genetic assessment of the English bulldog. *Canine Genetics and Epidemiology*.

[22] Ossowski A., Piatek J., Parafiniuk M. (2017). Genetic variation of 15 autosomal STRs in a population sample of Bedouins residing in the area of the Fourth Nile Cataract, Sudan. *Anthropologischer Anzeiger*.

[23] Peakall R., Smouse P. E. (2012). GenAlEx 6.5: genetic analysis in excel. population genetic software for teaching and research-an update. *Bioinformatics*.

[24] Won S. Y., Kim Y. C., Do K. (2020). Absence of strong genetic linkage disequilibrium between single nucleotide polymorphisms (SNPs) in the prion protein gene (PRNP) and the prion-like protein gene (PRND) in the horse, a prion-resistant species. *Genes*.

[25] Tavtigian S. V., Greenblatt M. S., Lesueur F., Byrnes G. B. (2008). In silico analysis of missense substitutions using sequence-alignment based methods. *Human Mutation*.

[26] Gharahkhani P., O’Leary C. A., Kyaw-Tanner M., Sturm R. A., Duffy D. L. (2011). A non-synonymous mutation in the canine Pkd1 gene is associated with autosomal dominant polycystic kidney disease in bull terriers. *PLoS One*.

[27] Houpt K. A. (2007). Genetics of canine behavior. *Acta Veterinaria Brno*.

[28] Vaysse A., Ratnakumar A., Derrien T. (2011). Identification of genomic regions associated with phenotypic variation between dog breeds using selection mapping. *PLoS Genetics*.

[29] Hu X.-S. (2005). Tension versus ecological zones in a two-locus system. *Theoretical Population Biology*.

[30] Marsden C. D., Ortega-Del Vecchyo D., O’Brien D. P. (2016). Bottlenecks and selective sweeps during domestication have increased deleterious genetic variation in dogs. *Proceedings of the National Academy of Sciences of the United States of America*.

[31] Proschowsky H. F., Olsen J. B., Jepsen B., Fredholm M. (2003). Evaluation of the present breeding programme against copper toxicosis in Danish Bedlington terriers. *Animal Genetics*.

[32] Lindblad-Toh K., Wade C. M., Karlsson E. K., Jaffe D. B., Kamal M., Clamp M. (2005). Genome sequence, comparative analysis and haplotype structure of the domestic dog. *Nature*.

[33] Sutter N. B., Eberle M. A., Parker H. G. (2004). Extensive and breed-specific linkage disequilibrium in *Canis familiaris*. *Genome Research*.

[34] Short A. D., Kennedy L. J., Barnes A. (2007). Hardy weinberg expectations in canine breeds: implications for genetic studies. *Journal of Heredity*.

[35] Jordana J., Piedrafita J., Sanchez A., Puig P. (1992). Comparative F statistics analysis of the genetic structure of ten Spanish dog breeds. *Journal of Heredity*.

[36] Ernst C., Hahnen E., Engel C. (2018). Performance of in silico prediction tools for the classification of rare BRCA1/2 missense variants in clinical diagnostics. *BMC Medical Genomics*.

[37] Wu T. Z., Lo Y. R. (2000). Synthetic peptide mimicking of binding sites on olfactory receptor protein for use in ’electronic nose. *Journal of Biotechnology*.

[38] Benbernou N., Robin S., Tacher S., Rimbault M., Rakotomanga M., Galibert F. (2011). cAMP and IP3 signaling pathways in HEK293 cells transfected with canine olfactory receptor genes. *Journal of Heredity*.

