# Supplementary material for: Artificial Selection Drives SNPs of Olfactory Receptor Genes into Different Working Traits in Labrador Retrievers
Source: Genet Res (Camb). 2022 Feb 2;2022:8319396. doi: 10.1155/2022/8319396 (PMC8828343; doi:10.1155/2022/8319396)
Supplement: Supplementary Materials — Table S1. Primer sequences of 7 OR genes. [file 8319396.f1.doc]

**Table S1 Primer sequences of 7 OR genes**

| **Num** | **Gene** | **Chrom** | **Cluster name** | **Primer** | **Product**  **(bp)** | **TM**  **(℃)** |
| --- | --- | --- | --- | --- | --- | --- |
| 1 | *OR0006* | 21 | @29-31 | OR0006F: 5'-CTGGCAGGCAATGGCATTTT -3'  OR0006R: 5'-GCTGCCAACCCATACCAGAT -3' | 491 | 57 |
| 2 | *OR0007* | 18 | @47-51 | OR0007F: 5'-TCATTCCTTGGATGTGCAGC -3'  OR0007R: 5'-TGATGGGATTCAGCATGGGA -3' | 589 | 57 |
| 3 | *OR16C11* | 18 | @47-51 | OR16C11F: 5'- CTGTCTCTGGTGGACTTTGGT-3'  OR16C11R: 5'- TTTTTGATGCCCTTGAGCTGACT-3' | 513 | 57 |
| 4 | *OR08G02* | 18 | @47-51 | OR08G02F: 5'- CTGTAACACCCAAGGCACTG-3'  OR08G02R: 5'-AGATCAGTGGATTCAGCATTGGA-3' | 639 | 57 |
| 5 | *DOPRX09* | 33 | @8 | DOPRX09F: 5'- AGGTGGACTTCTCCATGCCA -3'  DOPRX09R: 5'- TGATCTTCTGACTGTGCAGCTA -3' | 360 | 57 |
| 6 | *OR7215* | 21 | @29-31 | OR7215F:5'-GACCTAGGTGTGTCCTTTTCCA-3'  OR7215R: 5'-AGCAAGTACAGAGCGCAGGA-3' | 470 | 57 |
| 7 | *OR04B06* | 21 | @32-33 | OR04B06F:5'-TCTGGTTACTTTGGCTGACC-3'  OR04B06R: 5'-ATGGAAAACTGAAGAGCCATGTAG-3' | 365 | 57 |
